# The power of cooperation

**DOI:** 10.1136/ejhpharm-2020-002338

**Published:** 2020-06-25

**Authors:** Hugo M van der Kuy

**Affiliations:** Clinical Pharmacy, Erasmus MC, Rotterdam 3015, The Netherlands

**Keywords:** clinical pharmacy, health policy, public health, research methodology, internal medicine

COVID-19 has changed the world rapidly in ways we would never have imagined at the end of 2019. This has been described already earlier in this journal.^1^ Research however appears to be blooming like weeds in spring. It comes from everywhere, grows everywhere and sometimes it becomes important and sometimes its destiny is an early death.

China faced the virus 2–3 months ahead of Europe, which might have given them a window of opportunity to set the example.

Unfortunately, most of the research published until now from the ‘COVID-china’ is relatively small, a single-arm with no control group, no randomisation and often from a single centre.

This is unfortunate as it has led to the treatment of thousands of people on assumptions that were not scientifically based as we normally require, for example, the treatment with chloroquine.

Overwhelmed by the number of patients admitted to the hospitals one of the most important tenets of medicine was hampered: ‘primum no nocere’, first do no harm. Apart from the therapeutic problems, the world has been faced with logistical challenges at a time when most logistical transport were shutting down. This has lead in several countries to shortages of essential ICU medicines. The response of some people is to become egoistic: first my hospital, then the area of our hospital followed by our country and then the rest of Europe and finally the rest of the world. This has led to a reaction of countries to close their borders for the export of certain medicines. Nine leading academic hospitals from the EUHA (European University Hospitals Alliance) in Europe have published a letter to urge the European committee to take the lead and think Europe-wide instead of a self-serving reflex.^2^ It is however difficult to let rational thoughts of cooperation rise above animal instincts to get everything arranged right for yourself.

Back to the area of therapeutic research, I hope that the overwhelming amount of research that has started in several countries all over Europe does not lead to unfinished trials that are not sufficiently powered to solve key therapeutic questions. The future will tell us if we in Europe did better than China. In this journal a protocol article is published which describes an observational study across Europe. At the moment this Editorial is written, no data are currently available. However, the study is supported by 13 countries in Europe and more than 60 hospitals.

Apart from any therapeutic outcomes, the enthusiastic reactions from all over Europe, by mail, LinkedIn or telephone have provided an energy boost that can be compared with winning an Olympic medal! It has strengthened my belief in the power of cooperation. Together we are strong.^3^
